# Method development and clinical validation of LAMP-CRISPR/Cas12a for rapid detection of respiratory pathogens in children

**DOI:** 10.3389/fped.2025.1533100

**Published:** 2025-04-11

**Authors:** Siyan Zhou, Xin Zhao, Fanzheng Meng

**Affiliations:** ^1^ Pediatric Department of Respiration, The First Bethune Hospital of Jilin University, Changchun, China; ^2^ Department of Pediatric Respiratory, Children's Medical Center, The First Hospital of Jilin University, Changchun, China

**Keywords:** respiratory tract infections, LAMP-CRISPR/Cas12a, *streptococcus pneumoniae*, *mycoplasma pneumoniae*, molecular diagnostics

## Abstract

**Background:**

Respiratory tract infections pose a substantial health burden, particularly among pediatric populations globally. The timely and accurate identification of pathogens such as *Streptococcus pneumoniae* (SP) and *Mycoplasma pneumoniae* (MP) is critical for effective clinical management.

**Methods:**

In this study, a novel diagnostic approach combining loop-mediated isothermal amplification (LAMP) with CRISPR-Cas12a technology was developed for detecting SP and MP in clinical respiratory samples. A total of 23 specimens, including bronchoalveolar lavage fluid and nasopharyngeal swab samples, were assessed to evaluate the feasibility and performance of the method. After nucleic acid extraction, samples underwent LAMP amplification followed by CRISPR-Cas12a-mediated fluorescence detection.

**Results:**

The LAMP-CRISPR/Cas12a method demonstrated high sensitivity and specificity for SP detection. It exhibited excellent sensitivity for SP and promising specificity for MP. Comparative analysis with standard diagnostic methods highlighted its potential to enhance diagnostic accuracy and efficiency. The assay provided results within 1 h, which is suitable for rapid point-of-care testing.

**Conclusion:**

The integrated LAMP-CRISPR/Cas12a approach represents a significant advancement in detecting respiratory pathogens in clinical settings. It offers a rapid, sensitive, and specific diagnostic tool for identifying SP and MP, which is crucial for guiding precision therapies and improving patient outcomes. Future research aims to optimize assay sensitivity, streamline workflow to minimize contamination risks, and expand its detection scope so that other types of pathogens and mutation resistance genes can be detected. This molecular diagnostic strategy holds promise for the management of respiratory infections by enabling early and precise pathogen identification.

## 1 Introduction

Currently, childhood respiratory tract infections stand out as one of the most prevalent infectious diseases that pose a significant threat to children's health, especially in developing countries. Respiratory infections contribute to over one million deaths annually among children, underscoring their high incidence and severity in pediatric patients (1). These infections are primarily caused by a variety of pathogens, including bacteria [e.g., *Streptococcus pneumoniae* (SP) and *Haemophilus influenzae*], viruses (e.g., respiratory syncytial virus and influenza virus), and other microorganisms [e.g., *Mycoplasma pneumoniae* (MP)], with increasing concerns regarding their antimicrobial resistance (2–4).

Early and accurate pathogen detection remains pivotal in clinical practice. Traditional microbiological methods, such as bacterial culture, serological antibody testing, and polymerase chain reaction (PCR) technology, suffer from limitations including long turnaround times, an extended detection window, and high equipment costs (5, 6). Especially in the context of frequent pathogen mutations and rising antimicrobial resistance, there is a pressing need for a faster, more sensitive, and cost-effective diagnostic approach to guide clinical decision-making procedures (7).

In recent years, the rapid advancement of isothermal amplification technologies, particularly loop-mediated isothermal amplification (LAMP), has offered a promising solution. Unlike conventional PCR techniques, LAMP does not rely on thermal cycling and can rapidly amplify nucleic acid sequences under simple isothermal conditions (8). Coupled with downstream technologies such as real-time fluorescence detection, LAMP has demonstrated potential in early diagnosis across various diseases, especially in resource-limited settings and point-of-care testing (9).

Simultaneously, the application of CRISPR-Cas systems has revolutionized pathogen detection technology. Originally utilized for genome editing, the specific DNA recognition and cleavage capabilities of CRISPR-Cas systems have been extensively applied to the specific detection of pathogens. Particularly, *Cas12a* (formerly known as *Cpf1*), as one type within Class 2 of the CRISPR-Cas system, can recognize target DNA sequences when combined with specific CRISPR RNA (crRNA) and then activate the cleavage activity for nonspecific DNA sequences, thereby enabling detection and signal output from nonspecific sequences (10). This CRISPR-Cas12a-based technology has been demonstrated to rapidly detect various nucleic acids of pathogens with a high accuracy, showcasing its broad potential in clinical practice.

Against this backdrop, this study aimed to integrate LAMP and CRISPR-Cas12a technologies to establish a novel method for detecting respiratory pathogens in children. This method utilized LAMP technology for rapid amplification of target DNA sequences, followed by specific recognition and cleavage using CRISPR-Cas12a technology, with a fluorescent signal output for qualitative detection. The p1 gene of MP encodes a major adhesin protein essential for microbial attachment to host epithelial cells, making it a reliable target for pathogen identification. Similarly, the *cpsA* gene of SP is involved in bacterial capsule synthesis, a key virulence factor, and is widely used for pneumococcal detection. We evaluated the performance of an integrated LAMP-CRISPR/Cas12a assay for detecting common pathogens such as SP and MP in clinical respiratory samples by comparing it with traditional microbiological diagnostic methods to assess its accuracy, sensitivity, and specificity in clinical diagnosis.

Furthermore, this research explored the principles, current applications, and future trends of LAMP-CRISPR/Cas12a technology in the detection of childhood respiratory pathogens, contributing new theoretical and practical methods to improve the diagnosis and treatment of pediatric infectious diseases.

## 2 Materials and methods

### 2.1 Study population

We selected 20 pediatric patients admitted to the Department of Pediatric Respiratory Medicine, First Hospital of Jilin University, between January 2021 and March 2021. These patients were diagnosed with respiratory tract infections and underwent pathogen-specific diagnostics. After excluding samples that did not meet the detection standards, a total of 23 respiratory samples were collected, including 16 bronchoalveolar lavage fluid (BALF) samples and 7 nasopharyngeal swab (NPS) samples. All 23 samples were used in the validation of the LAMP-CRISPR/Cas12a assay to assess its clinical performance. The age of the patients ranged from 3 years to 13 years. All patients received treatment according to the “Guidelines for the Management of Community-acquired Pneumonia in Children (2013 edition)” (11), and comprehensive clinical, radiological, and laboratory investigations were conducted.

### 2.2 Inclusion and exclusion criteria

The inclusion criteria were as follows: (i) All pediatric patients diagnosed with pneumonia or bronchopneumonia according to the criteria outlined in the “Guidelines for the Management of Community-acquired Pneumonia in Children (2013 edition)” (11); (ii) patients who underwent bronchoscopy with bronchoalveolar lavage following procedures compliant with the “Guidelines for Pediatric Flexible Bronchoscopy in China (2018 edition)” (12); (iii) age greater than 3 years.

The exclusion criteria were as follows: (i) Patients with genetic metabolic disorders, congenital diseases, severe immunodeficiency diseases, or a history of immune or hematological system disorders; (ii) patients with moderate-to-severe organ dysfunction, defined as abnormal liver function tests (alanine aminotransferase or aspartate aminotransferase >3× upper limit of normal), renal insufficiency (estimated glomerular filtration rate <60 ml/min/1.73 m^2^), or cardiac dysfunction (New York Heart Association class III–IV or significant echocardiographic abnormalities); (iii) patients with chest wall deformities, tracheal, bronchial, or pulmonary developmental abnormalities; (iv) patients with active tuberculosis infection.

These criteria were applied rigorously to ensure the consistency and reliability of the study cohort, with adherence to ethical standards and clinical guidelines for research involving human subjects.

### 2.3 Clinical microbiological diagnostic criteria

Patients admitted to the First Hospital of Jilin University underwent a series of microbiological examinations. These included serological testing for MP antibodies using a chemiluminescence assay, blood culture, BALF culture, and nucleic acid detection of respiratory pathogens in BALF utilizing isothermal amplification techniques such as LAMP. A positive result in any of these tests, including MP antibodies (IgM positive or IgG > 300 U/L), confirmed the presence of pathogen infection (MP or SP).

### 2.4 Ethics approval

The study protocol was approved by the Medical Ethics Committee of the hospital (Approval No. AF-IRB-030-06). Informed consent was obtained from the guardians of all 20 patients who underwent bronchoscopy, ensuring compliance with ethical standards for medical research involving human subjects. All clinical data, imaging examinations, and laboratory results were collected and analyzed with full adherence to patient confidentiality and ethical guidelines.

### 2.5 Experimental materials, instruments, and specimen collection methods

This study utilized commercially available reagents for LAMP amplification, the CRISPR-Cas12a reaction, and nucleic acid extraction as well as standard laboratory instrumentation for fluorescence detection. Samples were sent to WillingMed Technology Co., Ltd. (Beijing, China) for metagenomics next-generation sequencing (NGS) using second-generation high-throughput DNA sequencing methods. All oligonucleotides, including isotheral amplification primers, modified sequences, and gene sequences, were synthesized by Sangon Biotech (Shanghai, China). A detailed list of reagents, full sequences of the primers and crRNA, and instruments is provided in the Supplementary Materials.

Specimens were collected as follows: NPSs were obtained using standard procedures, with the swab head immersed in a 5-ml collection tube containing 2 ml of sampling solution (including biological buffer and nucleic acid extraction lysis solution) and discarding the tail of the swab. BALF was collected according to the guidelines of the “Chinese Pediatric Flexible Bronchoscopy Procedure Guide (2018 edition)” (12), placed in sterile collection tubes, and stored at −20°C.

All oligonucleotides except for crRNA were resuspended in 1× TE buffer and stored at −20°C. crRNA was resuspended in nuclease-free water at 20 μM and stored at −80°C. Prior to experimentation, NPS and BALF samples were thawed to room temperature, mixed thoroughly to obtain a clear liquid, and processed accordingly.

### 2.6 Nucleic acid extraction of samples

**Figure 1:**
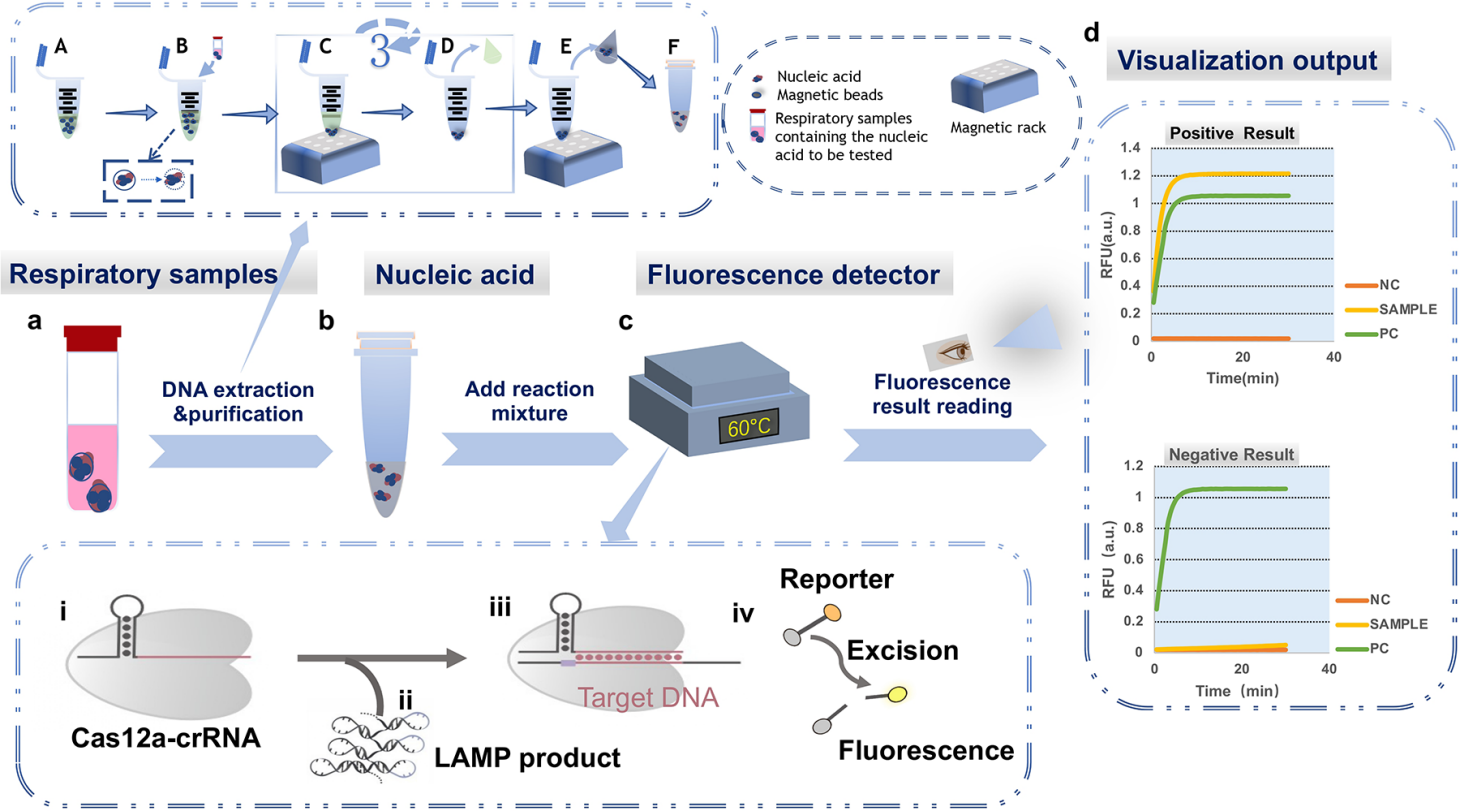
Integrated schematic of the LAMP-CRISPR/Cas12a diagnostic workflow, including nucleic acid extraction, reaction setup, and fluorescence detection. **(a)** Extraction and purification of clinical respiratory samples: **(A)** The reaction mixture, including lysis buffer, is premixed in a centrifuge tube; **(B)** Lysis: The respiratory sample to be tested (bronchoalveolar lavage fluid or nasopharyngeal swab collection fluid) is added to the reaction system. Cells are lysed, releasing intracellular DNA, RNA, and proteins into the liquid, and nucleic acids are adsorbed to the magnetic beads; **(C)** Washing: The centrifuge tube is placed on a magnetic rack, causing the nucleic acid-bound beads to adhere to the bottom of the tube; **(D)** The supernatant is discarded, and steps **(c)** and **(d)** are repeated three times to purify the nucleic acids; **(E)** Elution: The nucleic acids are separated from the magnetic beads. The beads adhere to the bottom of the tube, and the supernatant containing the nucleic acids is transferred to a new centrifuge tube; **(F)** The purified nucleic acid samples are stored at −20°C. **(b)** Establishing the reaction system. **(c)** Executing the LAMP-CRISPR/Cas12a reaction process. (i) Formation of the CRISPR/Cas12a-crRNA binary complex; (ii) Addition of the LAMP reaction product containing the target sequence (target DNA) to the system; (iii) Formation of the CRISPR/Cas12a–crRNA–target DNA ternary complex, activating the Cas12a cleavage activity; (iv) Cleavage of the F-Q reporter gene, producing a fluorescent signal; **(d)** Plotting the curve based on the fluorescence readout (NC, negative control; PC, positive control; SAMPLE, sample); the upper graph shows the result for a positive sample, and the lower graph shows the result for a negative sample.

Prior to testing, the actual samples required nucleic acid extraction (Figure 1). Nucleic acid extraction was performed using the JIFA 502-B type magnetic bead-based nucleic acid purification kit, according to standard operating procedures. Initially, 20 μl of Proteinase K, 10 μl of FineMag Particles G, and 500 μl of Buffer MVN were premixed in a centrifuge tube and shaken thoroughly before use. Subsequently, 200 μl of the test sample was added to the tube, vortexed for 30 s, and left at room temperature for 10 min. The tube was then placed on a magnetic stand for 30 s until the magnetic beads were completely adsorbed, and the supernatant was carefully removed. After removing the tube from the magnetic stand, 700 μl of Buffer DWIP was added and mixed by shaking for 30 s. This purification process was repeated three times, and then the tube was opened and left at room temperature for 5–10 min. After removing the tube from the magnetic stand, 50–100 μl of RNase-free water was added, and the mixture was shaken at 70°C for 2.5 min. The tube was placed on the magnetic stand for 1 min. After complete absorption of the magnetic beads, the nucleic acid solution was carefully transferred to a new centrifuge tube. If not immediately used for experimentation, the solution was stored at −20°C.

### 2.7 The LAMP-CRISPR/Cas12a reaction process

**Figure 2:**
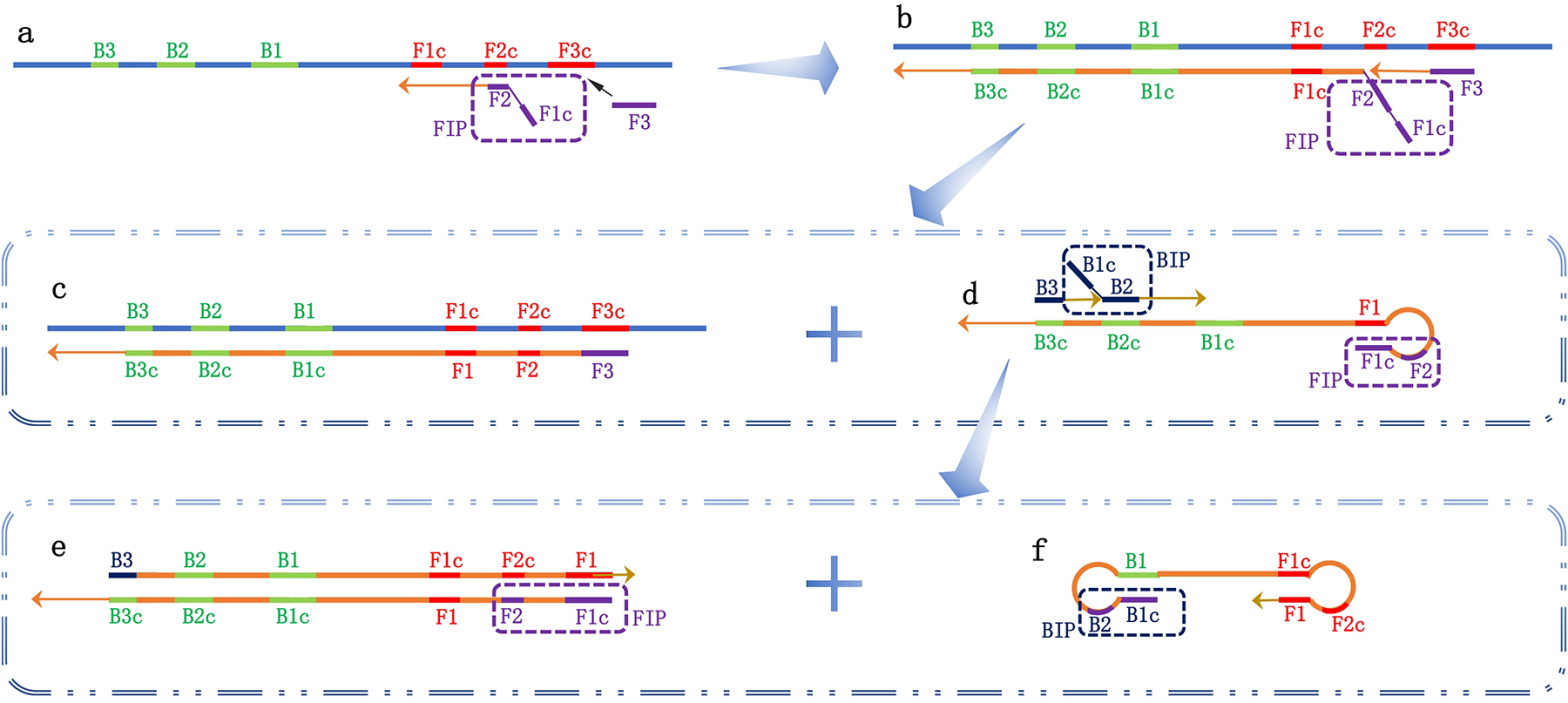
Schematic diagram of the loop-mediated isothermal amplification (LAMP) reaction. Four types of primers are used in the LAMP reaction: two inner primers [forward inner primer (FIP) and backward inner primer (BIP)] and two outer primers (F3 and B3). **(a)** The FIP primer recognizes the complementary sequence on the target nucleic acid, initiating the amplification; **(b)** The F3 primer recognizes the complementary sequence F3c, starting the first-strand exchange reaction and forming two reaction products, c and d; **(c)** After pairing with F3, a double-stranded template DNA complementary to the target nucleic acid is formed; **(d)** The complementary strand connected to FIP is released, and its two regions at the 5′ end (F1 and F1c) are complementary, forming a stem-loop structure. B3 and BIP recognize complementary sequences and begin the second-strand exchange reaction, forming two reaction products, e and f; **(e)** After pairing with B3, double-stranded DNA is synthesized, guided by the complementary strand connected to FIP; **(f)** The complementary strand connected to BIP is released, and the ends pair complementarily to form a dumbbell-shaped structure. This initiates the cyclic amplification of the LAMP reaction.

As shown in Figure 2, the LAMP reaction utilizes a total of four primers: two inner primers [forward inner primer (FIP) and backward inner primer (BIP)] and two outer primers (F3 and B3). The FIP includes F1c, a TTTT spacer, and a sequence complementary to F2c (F2). The BIP includes a sequence complementary to B1, a TTTT spacer, and B2 (B1c). The reaction cycles through these four primers to generate a stem-loop structure.

The primer mixture, containing 5 μM F3, 5 μM B3, 20 μM FIP, and 20 μM BIP, was prepared in advance. Each reaction system had a volume of 50 μl of 1× ISO buffer, including 6 μl of the synthetic target gene [MP *p1* gene (M21519.1) and SP *cpsA* gene (MK606437.1)] at different copy numbers, 4 μl of primer mixture, 4 μl of 4 mM dNTPs, 1 μl of 100 mM MgCl_2_, and 10 μl of 5 M betaine. The reaction mixture was heated at 95°C for 2 min and then cooled on ice for 2 min. Subsequently, 3 μl (8 U) of Bst 2.0 DNA polymerase was added to initiate the LAMP reaction. The reaction was incubated at 60°C for 90 min, followed by incubation at 80°C for 20 min to denature the DNA polymerase. The subsequent CRISPR/Cas12a reaction process was the same as that with the actual samples.

For the LAMP reaction setup using the actual samples, the primer mixture contained 5 μM F3, 5 μM B3, 20 μM FIP, and 20 μM BIP. Each reaction system had a volume of 25 μl of 1× ISO buffer, including 3 μl of extracted nucleic acid, 2 μl of primer mixture, 2 μl of 4 mM dNTPs, 0.5 μl of 100 mM MgCl_2_, and 5 μl of 5 M betaine. The reaction mixture was heated at 95°C for 2 min and then cooled on ice for 2 min. Subsequently, 1.5 μl (8 U) of Bst 2.0 DNA polymerase was added to initiate the LAMP reaction. Each reaction mixture (20 μl) was placed in a LightCycler 96 instrument at 60°C for 30 min. The generated LAMP products underwent further reactions, where 1.5 µl of 20× EvaGreen was added to monitor DNA amplification in real time. Fluorescence readings were taken every 30 s and plotted over time for real-time quantitative analysis.

After incubation at 60°C for 90 min, the reaction was heated to 80°C for 20 min to deactivate the polymerase. Next, 2 μl of the LAMP product was added to a 20 μl reaction system containing 500 nM crRNA, 200 nM Cas12a, 200 nM reporter gene, and 2 μl of 10× Cas12a-ssDNA buffer II. Finally, the reaction mixture was placed in a LightCycler 96 instrument at 37°C for 30 min. Fluorescence readings were collected every 30 s and plotted over time for real-time quantitative analysis. The contents of the reaction system are listed in Table 1.

**Table 1:** CRISPR/Cas12a reaction system.

Reagent	Volume
Cas12a (200 nM)	4 μl
Buffer II 10×	2 μl
F&Q (200 nM)	0.5 μl
crRNA (500 nM)	0.5 μl
Nuclease-free H_2_O	11 μl

### 2.8 Detection result interpretation and quality control

The detection results were assessed based on the fluorescence curves observed in the FAM channel over a 30 min period. For valid experiments, the negative control should exhibit a flat curve with no exponential rise, while the positive control should display an exponential increase in fluorescence. If the positive control showed no fluorescence increase, the experimental data for that sample were deemed invalid, necessitating re-extraction and retesting.

The results were interpreted as follows: (1) A sample was interpreted as positive if the fluorescence curve in the FAM channel showed an exponential rise within 30 min, and the negative control curve remained flat. (2) A sample was interpreted as negative if the fluorescence curve in the FAM channel remained flat within 30 min, and the negative control curve showed no exponential rise. (3) If the sample's FAM channel fluorescence curve rose, but the negative control's curve also rose, laboratory aerosol contamination occurred. In such cases, the testing environment and reagents should be replaced, followed by sample retesting.

### 2.9 Detection process

The operational flowchart of the LAMP-CRISPR/Cas12a detection method, including nucleic acid extraction, reaction setup, and fluorescence detection, is depicted in Figure 1. First, the respiratory samples underwent nucleic acid extraction, and then they were added to the reaction system. The fluorescent signals were visualized in a nucleic acid amplification instrument, allowing for nucleic acid testing of the sample to be completed within 1 h.

### 2.10 Statistical analysis

Statistical analysis was conducted using SPSS 24.0 software. For normally distributed quantitative data, the results are presented as the mean ± standard deviation. Skewed data are represented by the median (interquartile range). Count data are expressed as frequencies and proportions.

The Kappa consistency test was performed using paired design contingency tables to evaluate the agreement between clinical microbiological diagnosis and the results of clinical nucleic acid testing compared to the LAMP-CRISPR/Cas12a detection method for the same pathogens (MP and SP). A Kappa value <0.20 indicated poor agreement between groups; 0.21–0.40 suggested fair agreement; 0.41–0.60 indicated moderate agreement; 0.61–0.80 suggested substantial agreement; and 0.81–1.00 indicated almost perfect or perfect agreement. A significance level of *P* < 0.05 was considered statistically significant.

The sensitivity, specificity, positive predictive value, and negative predictive value of the LAMP-CRISPR/Cas12a method were calculated using clinical microbiological diagnosis as the standard.

## 3 Results

### 3.1 Patient clinical information analysis

This study included a total of 20 patients, comprising 9 males (45%) and 11 females (55%). The age ranged from 3 years to 13 years, with a mean age of 11.25 ± 5.48 years. The hospitalization duration ranged from 1 day to 28 days, with an average of 11.25 days. The time from symptom onset to hospital admission was 4–35 days, with an average of 13.30 days. Bronchoscopy was performed within 0–7 days of admission, with an average of 2.15 days. Other general clinical data are presented in Table 2.

**Table 2:** General clinical data of patients.

Category	Type	Cases (%)
Sex	Male	9 (45)
	Female	11 (55)
Symptoms	Fever	13 (75)
	Dry cough	10 (50)
	Productive cough	10 (50)
	Wheezing	1 (5)
	Others	5 (25) (Chest pain: 1 case, Abdominal pain: 4 cases)
Imaging abnormalities	Pneumonia	19 (95)
	Lung consolidation	2 (10)
	Atelectasis	9 (45)
	Pleural effusion	6 (30)
	Lung abscess	4 (20)
	Bronchiectasis	1 (5)
Pulmonary complications	Atelectasis	4 (20)
	Pleural effusion	2 (10)
	Lung abscess	1 (5)
	Bronchiolitis obliterans	2 (10)
Other complications	Extrapulmonary infection	2 (10)
	Organ damage	2 (10)
Medication history outside of the hospital	Macrolide antibiotics	12 (60)
	β-lactam antibiotics	13 (65)
	Steroids	2 (10)

### 3.2 LAMP-CRISPR/Cas12a detection method feasibility

**Figure 3:**
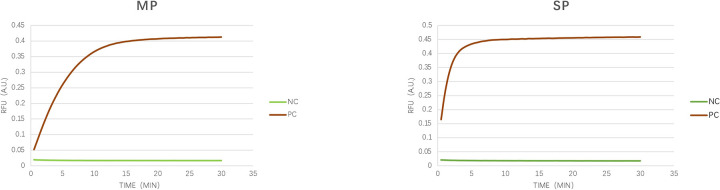
Verification of detection feasibility. As illustrated in the figure, we verified the feasibility of the CRISPR/Cas12a detection technology using synthetic target genes from MP (*p1* gene, M21519.1) and SP (*cpsA* gene, MK606437.1). The amplification curves displayed a typical sigmoid shape, whereas the negative control remained unchanged over the 30-minute duration. NC, negative control; PC; synthetic sequence).

The feasibility verification experiment of the LAMP-CRISPR/Cas12a detection method for MP and SP detection is shown in Figure 3, with the fluorescence curve illustrated. The results demonstrate that the genomic DNA fluorescence curves of the MP and SP strains exhibited exponential amplification within 15 min, characterized by an S-shaped amplification curve. In contrast, the negative control showed no change within 30 min, indicating that this technique can achieve rapid and accurate detection of these two pathogens.

### 3.3 Pathogen detection rates

Respiratory samples from 20 patients were analyzed to assess the pathogen detection rates using various methods. MP was detected positively in 18 cases, with 14 cases (70%) showing single MP infections, 2 cases (10%) with MP co-infections involving SP, and another 2 cases (10%) involving MP and *Haemophilus influenzae*. Clinical nucleic acid testing and LAMP-CRISPR/Cas12a methods each detected MP in 44.4% (8/18) of cases. For SP, it was identified in three cases, with all cases testing positive according to clinical nucleic acid testing and LAMP-CRISPR/Cas12a. Blood cultures were negative for SP in all 20 patients. This study also observed some additional clinical information such as antibiotic history, radiographic findings, and inflammatory biomarkers, which are detailed in the clinical data analysis.

### 3.4 Sensitivity and specificity of LAMP-CRISPR/Cas12a

A total of 23 qualified respiratory samples were collected, including 16 BALF samples and 7 NPS samples. When the LAMP-CRISPR/Cas12a detection results were consistent with the clinical pathogen diagnostic results for the same pathogen in the same specimen, it was considered a dual-positive result. There were nine dual-positive cases for MP detection and four for SP detection. The sensitivity, specificity, positive predictive value, and negative predictive value are shown in Table 3.

**Table 3:** Sensitivity and specificity of the LAMP-CRISPR/Cas12a detection method for *Mycoplasma pneumoniae* (MP) and *Streptococcus pneumoniae* (SP).

Pathogen	Dual positive (cases)	Dual negative (cases)	Sensitivity	Specificity	Positive predictive value	Negative predictive value
SP	4	13	100.00%	72%	50%	100%
MP	9	2	42.86%	100%	100%	14.29%

### 3.5 Consistency between the clinical nucleic acid testing/pathogen diagnostic results and LAMP-CRISPR/Cas12a

As shown in Table 4, for SP detection, the results of this testing method were consistent with both the clinical pathogen diagnosis and the clinical nucleic acid testing results. For MP detection, this testing method was generally consistent with the clinical nucleic acid testing results, but not with the clinical pathogen diagnostic results.

**Table 4:** Comparison of consistency between the LAMP-CRISPR/Cas12a detection method and clinical pathogen diagnosis and clinical nucleic acid results.

Pathogen type	Reference detection technology	Positive samples (Cases, %)	LAMP-CRISPR/Cas12a- positive samples	Consistent sample count	Concordance rate	Kappa value (*P* value)	Consistency
MP	Clinical nucleic acid testing	8 (40%)	8 (40%)	8	100%	1.0 (0.000)	Basic consistency
	Clinical pathogen diagnosis	18 (90%)	8 (40%)	10	50%	0.138 (0.495)	No statistical significance
SP	Clinical nucleic acid testing	3 (15%)	8 (40%)	15	75%	0.42 (0.021)	Consistent
	Clinical pathogen diagnosis	3 (15%)	8 (40%)	15	75%	0.42 (0.021)	Consistent

MP, *Mycoplasma pneumoniae*; SP, *Streptococcus pneumoniae*.

### 3.6 Comparison of upper and lower respiratory tract samples

**Figure 4:**
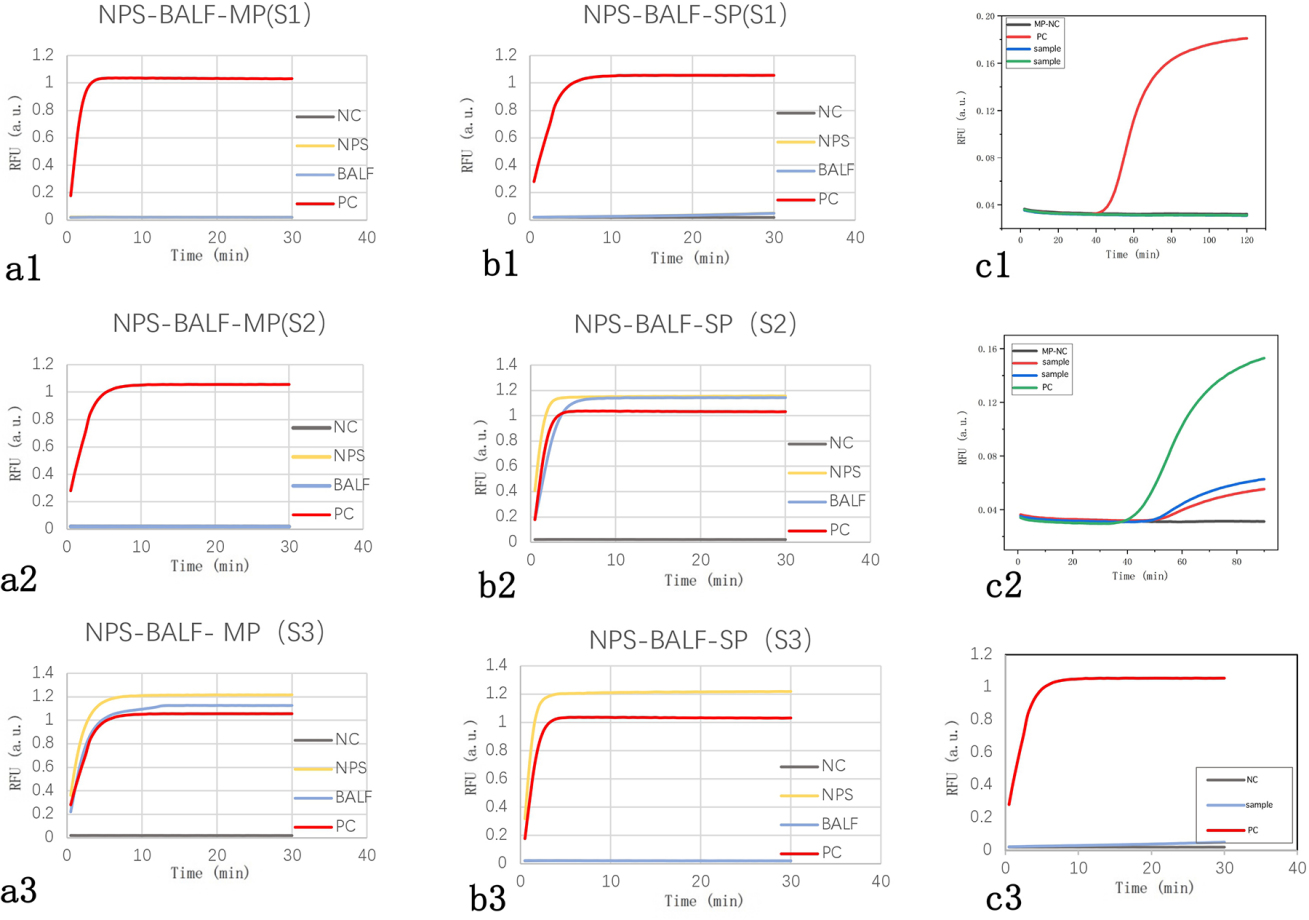
Fluorescence curves of actual clinical samples. (A1–3) Fluorescence curve comparisons of upper and lower respiratory tract samples for three cases of *Mycoplasma pneumoniae* infection, showing consistent results: two negative (S1, S2) and one positive (S3). (B1–3) Fluorescence curve comparisons of upper and lower respiratory tract samples for three cases of *Streptococcus pneumoniae* infection, with consistent results in two cases (S1 negative, S2 positive) and one inconsistent case (S3 positive in NPS, negative in BALF). (C1) A typical negative fluorescence curve for LAMP. (C2) Fluorescence curve showing a small s-shaped LAMP amplification curve. (C3) Fluorescence curve for the same sample as in C2 using the LAMP-CRISPR/Cas12a reaction. NC, negative control; PC, positive control; NPS, nasopharyngeal swab; BALF, bronchoalveolar lavage fluid.

Among the 20 patients, 3 underwent synchronous testing of the upper and lower respiratory tract samples. As shown in Table 5, there was one case (S3) where the NPS sample tested positive for SP while the BALF sample tested negative. The results from the other patients’ upper and lower respiratory tract samples were consistent. Additionally, among the 23 samples tested, the LAMP results were completely consistent with the LAMP-CRISPR/Cas12a results. However, the fluorescence curve trends in some upper respiratory tract samples tested using LAMP technology showed discrepancies compared to LAMP-CRISPR/Cas12a (Figure 4).

**Table 5:** Comparison of consistency between upper and lower respiratory tract sample results.

Patient number	Clinical Pathogen diagnosis	Laboratory test results	NPS	BALF
S1	MP	MP	−	−
		SP	−	−
S2	MP, SP	MP	−	−
		SP	+	+
S3	MP	MP	+	+
		SP	+	−

MP, *Mycoplasma pneumoniae*; SP, *Streptococcus pneumoniae*; BALF, bronchoalveolar lavage fluid; NPS, nasopharyngeal swab.

### 3.7 Further validation using NGS in samples with discordant clinical and LAMP-CRISPR/Cas12a results

We selected three cases with discrepancies between the clinical and LAMP-CRISPR/Cas12a testing results for further validation using NGS. The detailed information and NGS results are shown in Table 6. The results indicate that the sample N2 tested positive for SP by NGS, validating the positive result detected by our method, while it was not detected by the conventional clinical method. Samples N1 and N3 tested positive for MP by NGS, suggesting possible false-negative test results by our method compared to clinical microbiology (positive antibody, negative nucleic acid).

**Table 6:** Information and results of next-generation sequencing (NGS) samples.

Sample number	Diagnosis	Clinical pathogen diagnosis	LAMP-CRISPR/Cas12a detection results	NGS pathogen Genus/Species/Sequence count
N1	Severe pneumonia, necrotizing pneumonia	MP	No positive detection	*Streptococcus*/*Streptococcus pyogenes*/356947; *Mycoplasma*/*Mycoplasma pneumoniae*/3
N2	Pneumonia	MP, *Haemophilus influenzae*	SP-positive	*Corynebacterium*/*Corynebacterium congestum*/14467; *Haemophilus*/*Haemophilus influenzae*/3873; *Streptococcus*/*Streptococcus pneumoniae*/56
N3	Pneumonia	MP, SP	SP-positive	Measles virus/human influenza virus type 2/10; *Streptococcus*/*Streptococcus pneumoniae*/89074; *Mycoplasma*/*Mycoplasma pneumoniae*/22

MP, Mycoplasma pneumoniae; SP, Streptococcus pneumoniae.

## 4 Discussion

Respiratory tract infections are the most prevalent and deadly infections, largely due to delayed and inaccurate diagnosis and treatment (13). Pneumonia alone causes 14% of deaths in children under five years old (14), with bacteria playing a significant role alongside atypical pathogens in community-acquired pneumonia (15). Only one-third of children with bacterial pneumonia receive appropriate antibiotic treatment (16), emphasizing the critical role of accurate pathogen detection.

Advancements in molecular biology have shifted pathogen detection from traditional pathogen detection methods to specific antigen, antibody, and nucleic acid tests. Traditional microbiological methods (such as blood culture and BALF culture) are being replaced due to their inability to provide rapid and accurate clinical results, often leading to false-negative test results when antibiotics are used. One of the limitations of our study is the absence of a definitive gold standard for pathogen detection. While we compared our method with conventional microbiological and nucleic acid-based diagnostic approaches, each of these methods has inherent limitations. To improve diagnostic accuracy, future studies should consider incorporating NGS as a complementary reference standard, which may help to resolve discrepancies and provide a more comprehensive assessment of pathogen presence. This study highlighted the limited sensitivity of BALF culture, detecting SP in only one case.

PCR-based technologies like real-time PCR dominate clinical applications due to their specificity, while isothermal amplification methods are gaining attention for their simplicity and lower equipment requirements (17). LAMP is one of the more widely studied methods among these techniques. Coupling these methods with CRISPR-Cas technologies, particularly CRISPR/Cas12a, offers robust signal amplification and sensing capabilities (18). The high DNA specificity of CRISPR/Cas12a enables rapid and accurate diagnosis, which is critical for timely treatment initiation.

This study developed a novel detection method combining LAMP with CRISPR/Cas12a, leveraging the crRNA-CRISPR/Cas12a complex's DNA recognition specificity. Upon target recognition, Cas12a activates nonspecific cleavage activity, which in turn activates fluorescence via F-Q reporter gene cleavage. Previously, this method achieved high accuracy in detecting SARS-CoV-2 (19), and the current study proved to be sensitive (100%) and specific (72%) for SP detection in pediatric respiratory samples, surpassing those of conventional methods. This study innovatively applied this method for the combination of MP and SP.

The limit of detection (LoD) for MP in this study was 200 copies/μl, which falls within the range reported in clinical infections (typically 10^2^–10^6^ copies/μl, depending on disease severity). While this LoD is likely sufficient for detecting moderate-to-high burden infections, it may miss low-burden cases, particularly in early-stage infections or asymptomatic carriers. This limitation was observed in two MP-positive samples identified by NGS (N1 and N3), where detected MP sequences (3 and 22 copies) fell below the assay's LoD. Future studies will focus on enhancing assay sensitivity by optimizing primer designs, reaction conditions, and fluorescence detection thresholds. Additionally, semi-quantitative detection could be explored to provide a more detailed assessment of pathogen burden, which may aid in distinguishing active infections from colonization. This finding also confirms that this detection technology has a poor sensitivity for MP determination (42.86%), indicating that the lower limit of copy numbers for MP detection still needs further optimization and improvement. However, for moderate-to-high loads, this detection technology can produce clear positive results and demonstrate a high specificity, achieving 100% consistency with clinical nucleic acid detection techniques. In addition, in a study comparing the accuracy of LAMP and serological antibody for MP infection detection, the LAMP method was found to be faster rather than more sensitive. In this study, one patient was diagnosed with MP positivity through nucleic acid detection results rather than antibody detection results, and the LAMP-CRISPR/Cas12a detection method also indicated positive results. MP antibody detection refers to serological tests that measure the presence of IgM or IgG antibodies against MP in patient serum. IgM positivity or significant positivity of low-affinity IgG typically suggests recent infection. Despite its high sensitivity, serological antibody detection can be influenced by factors such as the timing of sample collection and individual immune response variations. In contrast, nucleic acid-based detection methods offer higher specificity and earlier diagnostic capability. Thus, the finding that one patient was diagnosed with MP positivity through nucleic acid detection results rather than antibody detection results may be due to the antibody detection results being influenced by multiple factors, including the detection window and individual immune function levels. Therefore, combining antibody detection and nucleic acid detection results for the etiological diagnosis of MP infection can improve the sensitivity and specificity of etiological diagnosis.

Although Table 3 demonstrates high specificity for MP and SP, the assay's performance against other common respiratory pathogens (e.g., *Haemophilus influenzae*, *Staphylococcus aureus*, and respiratory viruses) has not yet been fully evaluated. While the crRNA sequences were designed to minimize off-target effects, additional validation with an expanded panel of pathogens is required to rule out potential cross-reactivity. Future work will include broader specificity testing to confirm the robustness of this method in diverse clinical settings.

In the detection of respiratory pathogens in children, despite the limited reliability of upper respiratory tract samples, clinical practice tends to use noninvasive NPS for the initial diagnosis (20). However, SP colonizes the oral cavity and nasopharynx, affecting the accuracy of pathogen detection (21). For example, SP shares a high homology in its 16S rRNA gene with *Streptococcus viridans*, which also colonizes the oral cavity (22), posing identification challenges. Our study chose *cpsA* as a target for SP, demonstrating its potential as a specific detection site. SP can colonize the upper respiratory tract and causes infection only when immune barriers are compromised, necessitating differentiation between colonization and pathogenic strains in samples of the upper respiratory tract (23). Currently, there is no technology to accurately distinguish colonization strains, although NGS shows promise in this area, it is not yet feasible for routine detection. However, NGS is not yet feasible as a routine diagnostic tool. This study presented an example of SP-NPS-LAMP detection, showing a significantly reduced abundance of SP in the sample. Due to an insufficient BALF sample volume, NGS testing could not be performed, which is a limitation of this study.

A study by Nilsson *et al*. found that MP DNA can persist long-term in the nasopharynx of healthy individuals (24). Therefore, NPSs are suitable for qualitative MP detection but not for quantitative studies. However, during outbreaks of MP infections, asymptomatic carriers of MP are rare, and recent frequent regional outbreaks of MP infection highlight this trend. Despite challenges in identifying colonization strains and susceptibility of pharyngeal swab samples to contamination, this study attempted MP infection diagnosis using upper respiratory tract samples. Additionally, in patients with lower respiratory tract infections, the unnecessary use of macrolides and β-lactam antibiotics as imprecise treatments increases the risk of resistance (25). Previous studies have shown that SP (penicillin-binding proteins, mef gene, erm gene, mel gene, etc.) and MP (A2063G, A2064G, etc.) both have related resistance genes at resistance detection sites (26, 27). Over the past two years, the widespread prevalence of macrolide-resistant and hard-to-treat MP infections in Northeast China has highlighted an urgent clinical need for an accurate and rapid detection method for resistance genes. Detection of the above MP and SP resistance genes is one of the future research directions of this study.

This study presents a novel, rapid, and highly specific LAMP-CRISPR/Cas12a assay for detecting SP and MP in pediatric respiratory infections. By prioritizing speed, simplicity, and accessibility, this method has an enhanced diagnostic efficiency compared to traditional techniques and holds strong potential for point-of-care applications, especially in resource-limited settings. Future research will focus on multicenter validation, improved sensitivity, cross-reactivity testing, and potential integration into routine clinical workflows. Further refinement of semi-quantitative analysis and multiplex capabilities will also be explored to expand its diagnostic utility. With its high sensitivity and specificity for SP and MP, it is particularly suitable for point-of-care applications, where rapid decision-making is essential. Beyond its technical advantages, real-world implementation considerations, such as integration into clinical workflows, cost-effectiveness, operator training, and regulatory requirements, warrant further evaluation. Given its minimal equipment requirements and short turnaround time, this method holds strong potential for deployment in resource-limited settings, where conventional diagnostic tools may be less accessible. Future studies will include pilot implementations in clinical settings to assess its practicality and identify potential challenges in routine use. While our primary goal was to establish a fast and sensitive qualitative detection approach, we recognize that quantitative pathogen load analysis could provide additional clinical insights, such as infection severity assessment and treatment monitoring. Future studies will explore ways to refine fluorescence intensity interpretation and establish semi-quantitative threshold values, ensuring enhanced clinical decision-making without compromising the assay's speed and simplicity. Additionally, to strengthen clinical applicability, larger multicenter validation studies are needed. Given the heterogeneity of pediatric respiratory infections, expanding the sample size and study population will be crucial for confirming the robustness and generalizability of this method. Future research will focus on assessing its performance in diverse healthcare settings, ensuring its practicality in both well-equipped hospitals and resource-limited environments. However, we acknowledge that the relatively small sample size (*n* = 23) may limit the robustness and generalizability of our findings. A larger, multicenter validation study is necessary to confirm the clinical applicability of this method. Future research will focus on increasing the sample size and expanding the study population to enhance the reliability of our conclusions.

This study also has some additional limitations. First, although the technology shows good specificity and sensitivity for single-pathogen detection in laboratory conditions, it has not yet achieved single-tube detection of multiple pathogens within the same system. In real-world clinical settings, aerosol contamination and the presence of multiple nucleases may affect the accuracy and reliability of single-tube detection (28). Second, this study primarily focused on qualitative detection of pathogens. Although fluorescence signals were collected for real-time monitoring, it did not achieve fully quantitative detection. Further accumulation of samples is needed to establish a quantitative assessment of electronic results. Additionally, this study used a single-center retrospective analysis design and lacked strict gold standard references; moreover, it did not directly adjust treatment plans based on pathogen detection to improve patient outcomes. Future research requires more multi-center clinical validation to verify the effectiveness and practicality of LAMP-CRISPR/Cas12a technology in clinical applications. Finally, despite using magnetic bead nucleic acid extraction to reduce background contamination, further optimization of the experimental procedures and reagents is needed for the development of integrated, real-time detection systems. These improvements will help further promote the application of LAMP-CRISPR/Cas12a technology in clinical diagnostics, particularly in the early diagnosis and treatment of respiratory infections in children.

## 5 Conclusion

Our study developed and clinically validated a novel LAMP-CRISPR/Cas12a method for the rapid detection of childhood respiratory pathogens, thus addressing the urgent need for faster and more accurate diagnostic tools. This integrated approach demonstrated a robust accuracy and reliability, with the method showing excellent sensitivity for detecting SP and promising specificity for MP. Future research will focus on optimizing the workflow and reagents, designing related devices, and enhancing the sensitivity, ultimately establishing an integrated detection platform. This will broaden its application across diverse clinical settings, enabling true bedside and intraoperative point-of-care testing. In summary, this detection method offers advantages of speed, simplicity, and accuracy as well as holds the potential to establish a universal, portable, efficient, and precise point-of-care testing platform, making it a promising tool for detecting respiratory pathogens in children.

## Data Availability

The raw data supporting the conclusions of this article will be made available by the authors, without undue reservation.
