# Supplementary material for: Method development and clinical validation of LAMP-CRISPR/Cas12a for rapid detection of respiratory pathogens in children
Source: Front Pediatr. 2025 Apr 11;13:1533100. doi: 10.3389/fped.2025.1533100 (PMC12021640; doi:10.3389/fped.2025.1533100)
Supplement: Supplementary file 1 [file Table1.docx]

Supplementary Materials

Table S1. Sequence of oligonucleotides used in this work. Domains are separated by underscores.

| Name | Sequences (from 5ʹ to 3ʹ) | Label |  |
| --- | --- | --- | --- |
| MP-F3 | GGCCCGATTAATGGCTTGT | MP LAMP primers | |
| MP-B3 | GTTGAGTGGGCTGGCATT |  |  |
| MP-FIP | TCTGACTCCCCCCTTTCATCCCTACCCTGCTCGACACCTT |  |  |
| MP-BIP | AAGTGCAAACGACTTACCCGGTTAAGGAGGCAATTTTGGCGG |  |  |
| SP-F3 | GTTAGTGGAATTGACACCTATG | SP LAMP primers | |
| SP-B3 | GGTGTGAATGGACGAATC |  |  |
| SP-FIP | GTGGTCAAGAGGATTTTCTTGGTACTATTAGTTCGGTGTCGCG |  |  |
| SP-BIP | CCACGTGATGCCTATGTACCAAACTCCATAAATGCCCGC |  |  |
| MP-crRNA | UAAUUUCUACUAAGUGUAGAUCGUAUGUGACCCCCGUGA | CRISPR-Cas12a  MP crRNA | |
| SP-crRNA | UAAUUUCUACUAAGUGUAGAUUCUUUUUGAUUAUUUCCACCAUCU | CRISPR-Cas12a  SP crRNA | |

**Reagents and instruments**

10×isothermal buffer, Deoxynucleotide (dNTP) Solution Mix, RNase Inhibitor (Murine) and Bst 2.0 polymerase were purchased from New England Biolabs (Ipswich, MA, USA). 10×Cas12a- ssDNA Buffer II and LbaCas12a Nuclease were purchased from Novoprotein Scientific Inc. Isotheral amplification primers, modified sequences and crRNAs were synthesized by Sangon Biotech (Shanghai, China). The sequences of oligonucleotide used are presented in Table S1. Nucleic acid extraction Kit used for actual samples was purchased from Tiangen Biotech (Beijing, China). The supporting real-time fluorescence quantitative detector LightCycler 96 instrument (Roche Diagnostics GmbH, Germany), Applied Biosystems SimpliAmpTM PCR thermal cycler.
